# Efficacy of two different thiol-modified crosslinked hyaluronate formulations as vitreous replacement compared to silicone oil in a model of retinal detachment

**DOI:** 10.1371/journal.pone.0172895

**Published:** 2017-03-01

**Authors:** Sven Schnichels, Nele Schneider, Christine Hohenadl, José Hurst, Andreas Schatz, Kai Januschowski, Martin S. Spitzer

**Affiliations:** 1 Centre of Ophthalmology, University Eye Hospital Tübingen, Tübingen, Germany; 2 Croma Pharma GmbH, Leobendorf, Austria; 3 Department of Ophthalmology, University Medical Center Hamburg-Eppendorf (UKE), Martinistraße 52, Hamburg, Germany; Medizinische Universitat Graz, AUSTRIA

## Abstract

The efficacy of two novel artificial vitreous body substitutes (VBS) consisting of highly biocompatible thiolated cross-linked hyaluronic acid (HA)-based hydrogels in comparison to silicone oil in a model of retinal detachment was investigated. Pars plana vitrectomy (23G) was performed in the right eye of 24 pigmented rabbits. Retinal detachment of two quadrants was induced by creating a small retinotomy near the vascular arcade and injecting balanced salt solution (BSS) subretinally. The retina was reattached by injecting air, which was followed by increasing the infusion pressure, and the retinal tear was treated by endolaser photocoagulation. At the end of the procedure, the eye was filled either with 5000-cs silicone oil (after fluid air exchange) or the respective hydrogel (with two different viscosities). Follow-up examination included slit lamp examination, funduscopy, intraocular pressure measurements (IOP), optical coherence tomography (OCT) and electroretinogram (ERG) measurements. After a maximum follow-up of four weeks both eyes were removed, examined macroscopically, photographed, and prepared for histology. Of the eight rabbits that received silicone oil, seven (87.5%) developed a recurrent retinal detachment with pronounced proliferative vitreoretinopathy within the first two weeks after surgery. In contrast, in the hydrogel treated eyes, the retina stayed attached in the majority of the cases (73.3%). IOP and retinal morphology were normal as long as the retina remained re-attached. In conclusions, this model of retinal detachment, both thiolated crosslinked hyaluronate hydrogels showed superior efficacy when compared to silicone oil. These hydrogels have a promising potential as novel vitreous body substitutes.

## Introduction

Vitreous substitutes (endotamponades) such as silicone oils or gas tamponades are used to stabilize and reattach complicated cases of retinal detachment.[1] Because silicone oils are not biodegradable, a second surgery to remove the endotamponade is usually necessary. Gases and silicone oils may yield beneficial anatomical outcomes; however, refractive changes and risks associated with current tamponades (e.g. secondary glaucoma), and the potential hazards of the second surgery in the case of silicone oils, have triggered a search for new biocompatible vitreous body substitutes (VBS). Moreover, all commercially available tamponades have in common that they are hydrophobic and act through buoyancy and interfacial tension. However, the hydrophobic nature of these tamponades is responsible for the major disadvantage that a complete filling of the vitreous cavity is not possible. With all hydrophobic tamponades a fluidic space remains, allowing growth factors to accumulate. This often results in re-proliferation and re-detachment of the lower retina. Newly developed materials that are heavier than water, such as heavy silicone oil, unfortunately did not solve this problem but rather shifted it to the upper part of the retina. Potential alternatives to current VBS may be compounds that are hydrophilic, transparent, with a refractive index close to 1.33, and stable over a longer time. Furthermore, it may be advantageous if these substances show a higher viscosity and elasticity than the human vitreous body. Compared to gases and silicone oils, they will exhibit less surface tension and no buoyancy. Thus, a higher viscosity and a controlled, and moderate swelling of the VBS may serve as an alternative force to keep the retina reattached across retinal breaks until the retinopexy (cryopexy or photocoagulation) provides a permanent seal. In the present study a vitreous substitute based on cross-linked thiolated HA (tVBS) was evaluated. The thiol-modified polymer is able to build stable hydrogels by natural formation of disulfide bridges and thus does not require addition of chemical cross-linkers or other manipulation. The goal of this study was to evaluate the efficacy of a vitreous substitute based on tVBS in a model of retinal detachment in comparison to current standard treatment with silicone oil. Prior to their surgical use, the generated hydrogels were characterized with respect to optical and rheological properties and accordingly two tVBS hydrogels with differing viscosities were chosen for further evaluation.

## Material and methods

### Preparation of TCHA hydrogels

The test items are biodegradable, clear, viscous hydrogels, free of visible particles, homogenized and prepared as injectable gel implants, which have been steam sterilized. Formulations were prepared in physiological phosphate buffer (320 mOsmol/kg, pH 7.4) and contained either 2.2% (VBS strong) or 1% (VBS soft) HA. Crosslinking was achieved by inducing disulfide bridge formation in substituted thiol groups.

### Rheological analysis

A MCR101 rheometer equipped with a 50 mm cone-plate (CP50-1) measuring device (Anton Paar, Graz, Austria) was used for rotational and oscillatory rheological analysis. The linear viscoelastic range was determined by amplitude sweep measurements for each sample separately and the determined deformation γ was adjusted accordingly for the following frequency sweep measurements. Storage (G’) and loss modulus (G”) were determined and the ratio of viscous and elastic parts (Tan δ) calculated accordingly using the RheoPlus software (Anton Paar, Graz, Austria).

### Determination of refractive index

Before measurement, sample and device (Krüss refractometer AR 2008) were adjusted to 35°C ± 2°C. Double distilled water was measured as a control and the internal standard value adjusted accordingly. About 1 g of hydrogel was applied to the prism and the refractive index (RI) determined at 589 nm. In compliance with ISO16672:2003 RI was determined in addition at 546 ± 10 nm using a Carl Zeiss refractometer (CZ11401) and a LED lamp emitting at 546 nm.

### Animals and pre-operative evaluation

All animal procedures and methods were performed in accordance with the Statement for the Use of Animals in Ophthalmic and Vision Research of the Association for Research in Vision and Ophthalmology (ARVO), complied with institutional guidelines, and abided by EU and German law. All animal experiments were conducted under research permission AK1/14, granted by the Regierungspräsidium Tübingen, Germany, to M. S. S.

All experiments were performed on chinchilla bastard rabbits (ChBB:CH; Charles River Laboratories, Sulzfeld, Germany), after an acclimatization period of at least five weeks. Housing and husbandry conditions were compliant with EU-guidelines 2010/63/EU. The animals were housed in a pathogen free facility, one rabbit per two connected standard cages with unrestricted access to both cages (Tecniplast, Tecniplast Deutschland GmbH, Germany), with straw bedding, allowed free water and food access, kept in 12 hour light/dark cycles, 18°C and air humidity of 55% ± 10%.

Preoperative investigations on three groups, consisting of eight young (2 months old) pigmented rabbits, included ERG, OCT and examination of both eyes with the slit lamp and measuring the intraocular pressure.

ERGs, OCT and surgeries were performed under general anaesthesia using intramuscular injections of ketamine 10% (0.25 mg/kg) (Ketanest; Parke Davis, Berlin, Germany) and medetomidine hydrochloride (35 mg/kg) (Sedator; Eurovet, Bladel, Netherlands). All other examinations (except for IOP measurements) were performed under sedation with 25 mg/kg medetomidine. In addition, local anaesthesia with oxybuprocaine drops was applied throughout all the examinations, IOP measurement and surgeries (Novesine 0.4%; Novartis, Nürnberg, Germany). Post-surgery, all animals received 5mg/kg carprofen to reduce any possible pain. One animal, which died during the preexamination had a heart failure of unknown cause. The autopsy was performed together with a vet from the local animal protection office together with the vet participating in this study.

### ERG

ERG measurements were performed at baseline and one month after surgery with an Espion (Diagnosys LLC, Cambridge, UK). Animals were dark adapted for 30 minutes prior to ERG measurements. Pupils were dilated by tropicamide 0.5% (Mydriatikum Stulln, Stulln, Germany). To record the ERG, two contact lens electrodes and three needle electrodes (subcutaneous, one in the right upper lid, one in the left upper lid and one in the neck) were used. Methocel (2% methylcellulose; Omnivision GmbH, Puchheim, Germany) was applied regularly to the cornea to avoid exposure keratopathy. Measurements were performed only when acceptable impedance levels of less than 10 kΩ at 25 Hz (using the machine's built-in algorithm) were reached.

The dark-adapted ERG protocol consisted of three steps with increasing stimulus strengths from 0.003, 3 and 10 cd●s/m^2^ with a mixed white light (white 6500K) produced by a Ganzfeld stimulator (ColorDome; Diagnosys LLC, Cambridge, UK). All flashes were presented without background illumination and constant interstimulus intervals of 1 second for dim flashes and up to 30 seconds for bright flashes to maintain dark adaptation. Flash duration was 4 ms in all steps. Band-pass filtering was applied from 1.25 to 300 Hz. Averages ranged from 20 trials for dim flashes to two trials for bright flashes. Oscillatory Potentials were measured for 3 and 10 cd● s/m^2^.

Light-adapted ERGs were recorded after light adaptation with a background illumination of 30 cd/m2 (white 6500 K) for 3 minutes. Stimulus strength of 3 cd●s/m2 and 10 cd●s/m^2^ was chosen for single flash and 5, 10, 30 and 45 Hz for flicker responses. Twenty trials were averaged for single flash responses, and 30 trials for flicker stimulation.

Oscillatory potentials (OP) were analyzed after offline band-pass filtering with 75 to 300 Hz of ERG responses elicited by combined responses. OPs were calculated as area under the curve between the a-wave trough and the b-wave peak. Gaussian distribution of the data was ensured using a scotopic flash of 3 cd●s/m^2^ (Kolmogorov test p > 0.15)[2, 3].

### Optical coherence tomography

General swelling of the retina was examined at baseline and at one month, using high resolution OCT with the Spectralis® HRA+OCT device (Heidelberg Engineering, Heidelberg, Germany). During each examination, the visual streak was searched and retinal thickness was determined 3 mm centrally from the visual streak.

### Surgery

Pars plana vitrectomy was performed in the right eye. Surgery was performed by a single experienced surgeon (MSS) under general anaesthesia as described above. Retinal detachment of two quadrants was induced by creating a small retinotomy near the vascular arcade and by injecting subretinal balanced salt solution (BSS) subretinally. Retinal hemorrhage was induced by a circumscribed vascular breach in the periphery of the vascular arcade. The retina was reattached by increasing the infusion pressure and the blood was washed out until the bleeding stopped. The retinal tear was treated by endolaser photocoagulation. The surgeon did not know until this time-point of the procedure whether the eye was bound to receive silicone oil or hydrogel. At the end of the procedure the eye was either filled with 5000 cs silicone oil (ala Sil 5000, Alamedics GmbH & Co. Kg; Dornstadt, Germany) (after fluid air exchange) or the respective hydrogel (VBS strong or VBS soft) was injected into the vitreous cavity until egress of the tamponading agent through the opposite sclerotomy was noted. Finally, the sclerotomies and the conjunctival wounds were closed using 7.0 vicryl absorbable sutures (Ethicon, Norderstedt, Germany). After surgery, the right eye was re-examined at regular intervals and compared to the non-operated left eye by slit lamp examination, funduscopy and intraocular pressure measurements (the examination schedule is given in Table 1). The postoperative OCT and ERG were performed after one month. Unfortunately, one animal died during the pre-operative examination. Thus, the VBS soft group consisted of only seven rabbits. At the end of the study all rabbits were euthanized with an intracardial injection of T61 (Intravet, Germany) under general anesthesia as described before. During the study the animals were examined twice daily by a veterinary (N.S.). An early or humane end-point protocol included: severe toxicity of the vitreous substitute (monitored via ERG), irreversible damage to the eye: for example complete ablatio and scarring of the retina (monitored via OCT), severe infections: for example: endophthalmitis, loss of more than 20% body-weight, severe injuries or other spontaneous diseases. None of these end-points occurred in this study.

**Table 1:** Examination schedule.

Examination schedule					
	Preoperative examination	Day 1post-OP	Day 3 post-OP	Day 7 post-OP	1 month Post-OP
Weight	x	x	x	x	x
Slit lamp	x	x	x	x	x
IOP OS/OD	x	x	x	x	x
Funduscopy	x	x	x	x	x
ERG	x				x
OCT	x				x

### Histology and immunohistochemistry

Finally, both eyes were removed and processed for histology (H&E) and immunohistochemistry with anti-GFAP (glial fibrillary acidic protein) and anti-Brn3a (brain-specific homeobox/POU domain protein 3A) antibodies. Immunohistochemistry was only performed on areas in which the retina macroscopically appeared attached or relatively normal. The amount of GFAP-staining was graded by three independent examiners (NS, JH, SS) who did not know to which particular group the respective section belonged to. The staining was graded as mild (1), moderate (2), strong (3) or very strong (4) and after grading by the three independent researchers an average score was created for each section. Eyes with complete retinal destruction due to total PVR detachment were graded with 6.

Three representative Brn3a section (200x) of each case with attached retina were used for the determination of the number of ganglion cells.

## Results

Two different hydrogel formulations, VBS strong and VBS soft, were prepared from thiolated HA by induction of disulfide bridge formation. The generated VBS both revealed a refractive index of 1.34 at 589 nm and 1.32 at 546 nm, respectively. Rheological analysis with hydrogel pre-sheared by ejection from a 5 mL syringe through a 20-gauge needle resulted for VBS soft in a storage modulus (G’; ω = 1 1/s) of 150400 mPa and a loss modulus (G”; ω = 1 1/s) of 4693 mPa. Respective values for VBS strong were determined as G’ 663544 mPa and G” 41768 mPa reflecting the higher HA content and the more solid character of the latter formulation. Accordingly Tan δ of VBS strong was calculated as 0.0629 which was twice as high as the value calculated for VBS soft (Tan δ: 0.03121).

Injection force needed to extrude the hydrogel from a 5 ml syringe through a 20-gauge cannula was analyzed with a motorized device (Mecmesin, West Sussex, UK) and determined as 5.3 N for VBS soft and 14.2 N for VBS strong, respectively. These values are comparable to the force required to inject ophthalmic viscoelastic devices (OVDs) during cataract surgery.

In conclusion, the newly generated HA-based hydrogel formulations revealed all basic physical characteristics required for an application as vitreous substitute.

### Postoperative inflammation

Intraocular tamponades especially when instilled for the treatment of retinal detachment are often associated with intraocular inflammation. Thus, the amount of intraocular fibrin and anterior chamber cells was assessed frequently after retinal detachment repair. On the first postoperative day a moderate amount of fibrin was universally present in all animals. However, the resolution of fibrin took longest in the silicone oil group, with some fibrin persisting in half of the animals until the end of follow-up. In the VBS soft group the fibrin had cleared-off completely at the final follow-up, whereas in the VBS strong group mild amounts of fibrin could still be observed in 3/8 animals at the end of the study.

### Intraocular pressure (IOP)

**Fig 1:**
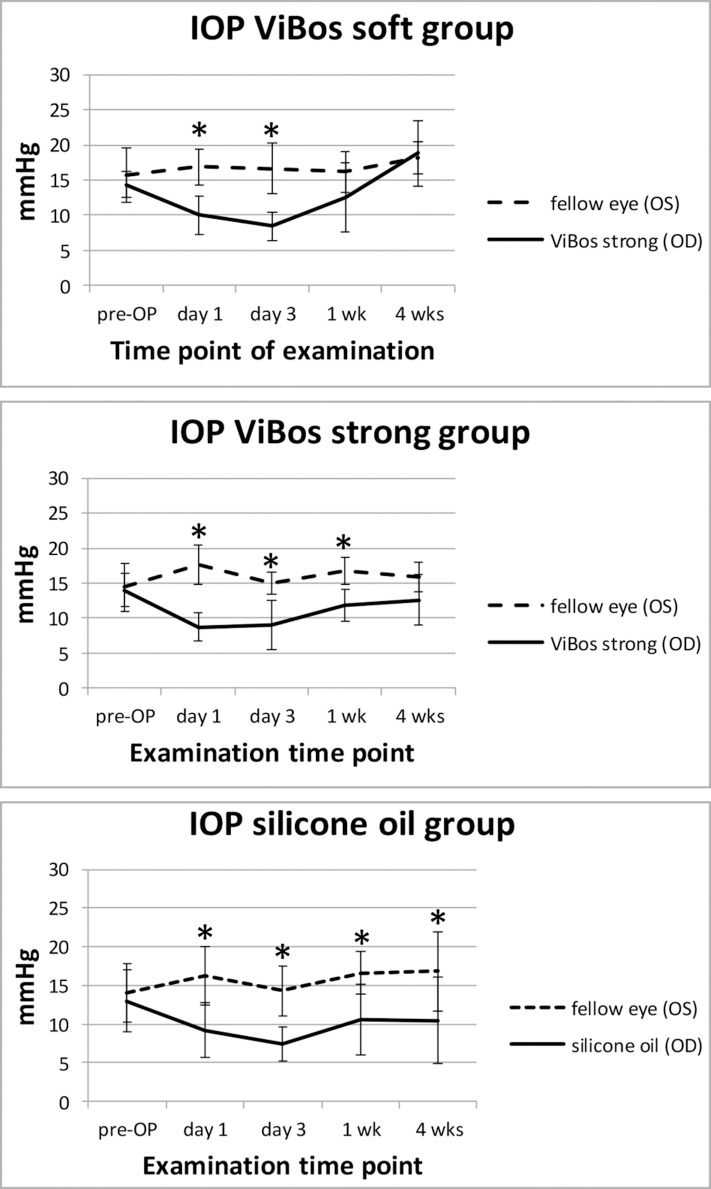
Intraocular pressure changes. Mean IOP levels for all groups. After surgery the IOP dropped but was still within clinically acceptable limits.* p<0.05 (Bonferroni adjusted t-test).

After instillation of silicone oil, VBS strong or VBS soft the IOP never reached worrisome high or low levels at any time point after surgery. Although the IOP after surgery was significantly lower in the operated eye than in the contralateral control eye of the respective group, the IOP was still well within clinically tolerable levels. The average IOP after silicone oil filling was significantly lower compared to the contra-lateral eye throughout the entire follow-up period (p<0.05; two-sided t-test, Bonferroni-corrected), whereas in both hydrogel groups the IOP returned to pre-operative levels at the end of the study **(Fig 1).**

### Anatomical results–retinal re-attachment

**Fig 2:**
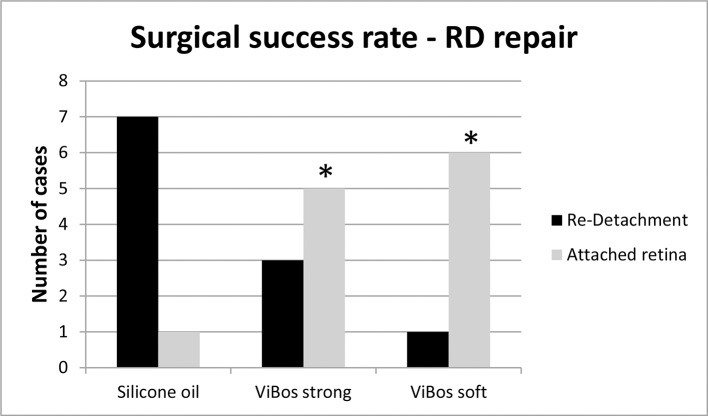
The rate of re-detachment was significantly lower when VBS soft or strong were used. The difference between the silicone oil treated eyes and the respective hydrogel group was statistically significant (p<0.05; Bonferroni-adjusted t-test).

Seven of eight rabbits that received silicone oil, developed a partial (one animal) or even total retinal detachment (six animals) with pronounced proliferative vitreoretinopathy within the first two weeks after surgery. In contrast in the VBS strong group only three of eight rabbits developed a new retinal detachment and two of these three retinal re-detachments were only partial detachments. No recurrence of retinal detachment was observed in the VBS soft group **(Fig 2).** After the last follow-up examination, both eyes were removed and prior to histology examined macroscopically. The difference between the silicone oil treated eyes and the respective hydrogel eyes was statistically significant (p<0.05; Bonferroni-adjusted t-test).

### Development of cataracts

Only a minority of eyes which received VBS soft (2 out of 7) or VBS strong (1 out of 8) developed cataracts. One of the cataracts in the VBS soft group was most likely due to iatrogenic lens touch during surgery. In contrast, the majority of silicone oil filled eyes (7 out of 8) had developed dense cataracts at the one month visit. None of the cataracts in the silicone oil group was due to iatrogenic factors. The difference between the silicone oil treated eyes and the respective hydrogel group was statistically significant (p<0.05; Bonferroni-adjusted t-test).

### OCT results

**Fig 3:**
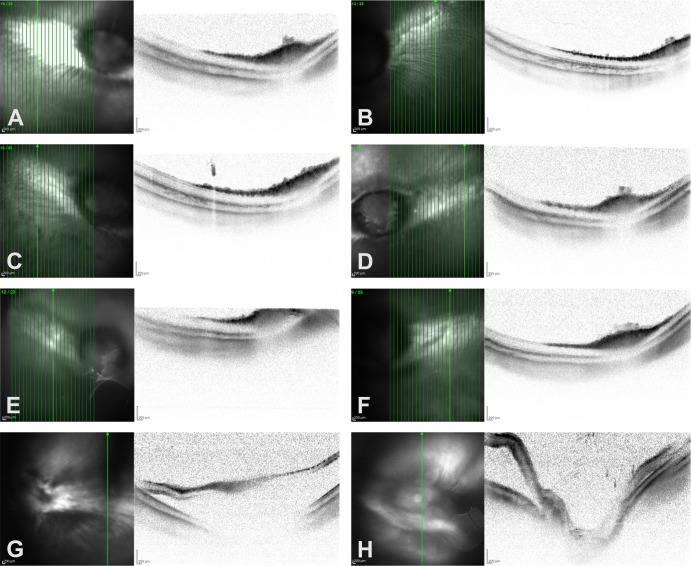
OCT was a valuable tool for in-vivo monitoring of retinal structure, showing attached and regular layered retina in cases operated with (A) silicone oil, (C) VBS strong or (E) VBS soft. The non-operated contralateral eyes of cases A, C and E are shown in B, D and F. (G and H) Partial detachments were observed by funduscopy and confirmed by OCT in two eyes that had received VBS strong.

Postoperative OCT at one month was nearly impossible when dense cataracts were present. Thus, especially in the silicone oil group in which the majority of the eyes had cataract at the final examination, follow-up OCT was mostly not possible. In contrast, in the majority of VBS soft or strong eyes OCT was feasible and showed attached and normally structured retina **(Fig 3).**

### ERG results

It could be shown that the data of the ERG measurements were normally distributed, which was important for the selection of the appropriate statistical tests for the evaluation of the ERG results. Thus, further analysis could be performed with a two-sided Student t-test. In order to ensure that there were no differences between right and left eye at baselines all ERG measurement of each group of the study were analyzed for differences between the right and the left eye. There was no statistically significant difference between the right and the left eye at baseline (Bonferroni-adjusted t-test).

The ERG of the silicone oil group was unchanged in only one rabbit, one was strongly reduced and in six rabbits the ERG was completely extinguished. The ERGs were in agreement with the anatomical findings: The eyes with a total retinal detachment had an extinct ERG, those with a partial detachment had a reduced ERG and the eye with a re-attached ERG a nearly normal ERG.

In contrast, the follow-up ERGs of the two hydrogel-groups were extinct in only two cases (each in one group). In the group with the more viscous formulation (VBS strong) the ERG was slightly reduced in five and strongly reduced in two animals one month after surgery for induced retinal detachment.

In the group with the softer formulation (VBS soft), the ERG was slightly reduced in three and moderately reduced in three animals one month after surgery for induced retinal detachment. It should be mentioned that retinal detachment and vitrectomy itself will cause some decrease of the ERG even when the retina is successfully re-attached.[4, 5] Moreover, vitreous substitutes such as silicone oil also have a slight but detectable insulating effect which also interferes with the ERG amplitudes.[6, 7] However, when the ERG under these circumstances is extinct, it is not due the respective vitreous substitute but due to the recurring and progressing retinal pathology (total retinal detachment) that leads to a complete loss of retinal function.

### Macroscopic examination

**Fig 4:**
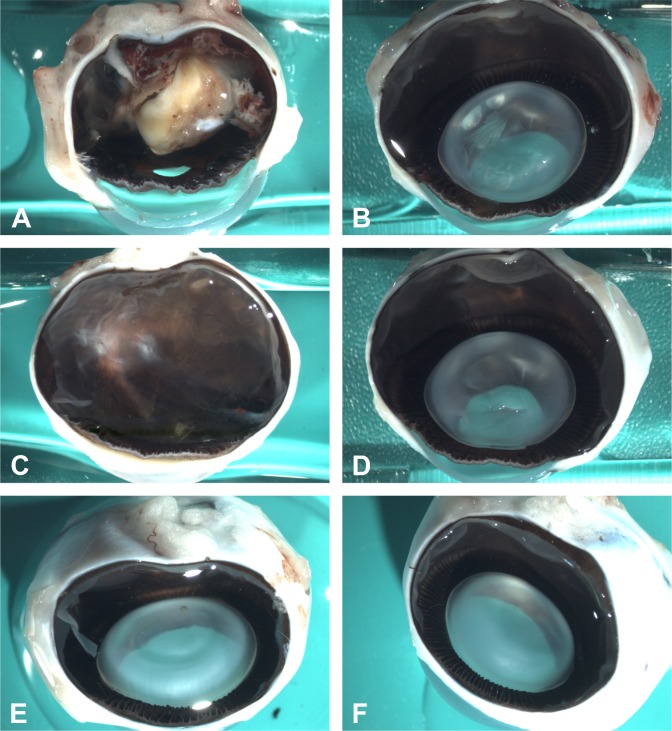
(A) Representative macroscopic pictures showing total retinal re-detachment with pronounced proliferative vitreoretinopathy in a case operated with silicone oil compared to attached retina in cases in which the retina was attached using (C) VBS strong or (E) soft. The non-operated contralateral eyes of each case are shown in B, D and F.

All retinal detachments that were visible on funduscopy were confirmed during macroscopic examination **(Fig 4)**

### Histologic examination

Eyes with re-detached retina uniformly developed pronounced proliferative vitreoretinopathy (PVR). In the silicone oil group only one operated eye showed normal retinal architecture on histology, five had a very severe PVR reaction and two a moderate amount of PVR.

**Fig 5:**
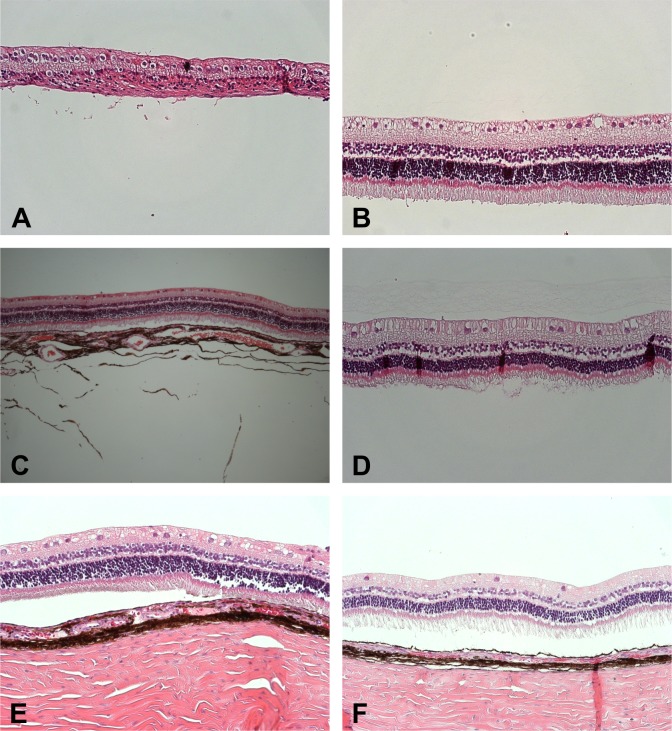
Representative microscopic pictures (H&E; 200x) showing retinal atrophy in a case of re-detached retina operated with (A) silicone oil compared to normally structured attached retina in cases in which the retina was attached using (C) VBS strong or (E) soft. The non-operated contralateral eyes of each case are shown in B, D and F.

In the VBS strong group six animals showed normal retinal architecture, the two other two had a moderate to pronounced PVR reaction. In the VBS soft group, two animals were unremarkable on histology. One animal showed a severe PVR reaction and four had small areas of focal retinal thinning but otherwise unremarkable retinal structure **(Fig 5).**

### GFAP and Brn3a expression

**Fig 6:**
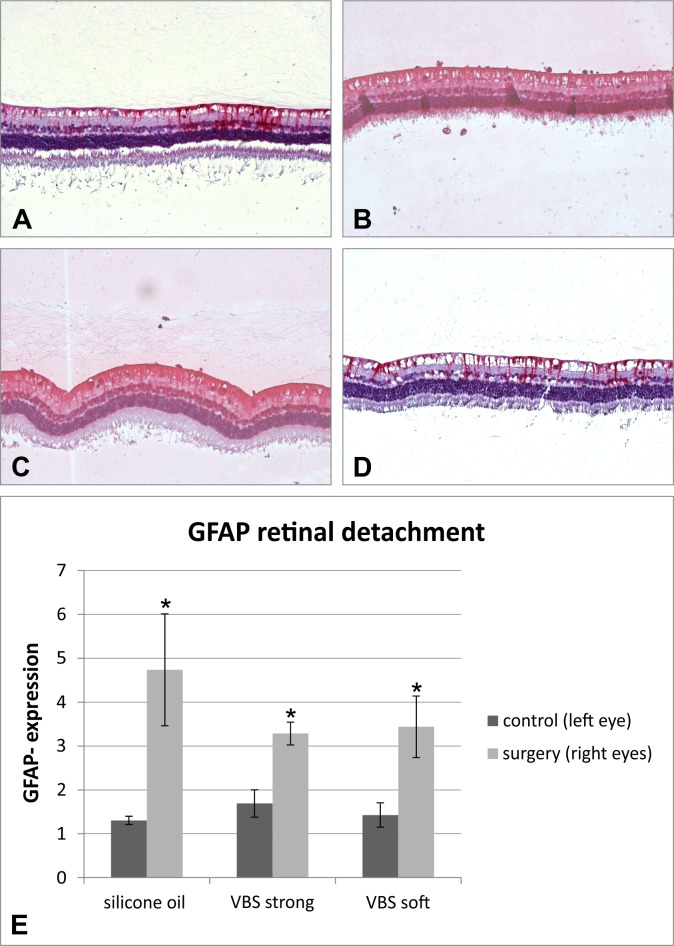
(E) GFAP expression was significantly up-regulated in all operated eyes compared to control (p<0.05; Bonferroni-adjusted t-test). Although the total amount of GFAP expression was highest in the silicone oil treated eyes, this difference was not statistically significant compared to the hydrogel groups. (A) Non-operated control eye with mild expression of GFAP (B). Eye operated with silicone oil showing strong GFAP expression. (C) Eye operated with VBS strong showing strong GFAP expression. (D) Eye operated with VBD soft showing moderate GFAP expression. (E) GFAP expression of all groups.

The expression of GFAP is a marker for glial activation and retinal fibrosis. Although GFAP expression was significantly up-regulated in all operated eyes the total amount of GFAP expression was highest in the silicone oil treated eyes (albeit not statistically significant compared to the hydrogel groups) **(Fig 6).**

The expression of the RGC-marker Brn3a was not significantly different in areas of attached retina between the operated and the control eye. This confirms the general good biocompatibility of all three employed tamponading substances (data not shown).

## Discussion

This study demonstrated that hydrophilic hydrogels based on thiolated cross-linked hyaluronate might be a potential alternative to silicone oils or gases for vitreoretinal surgery. Currently, only silicone oil is available as a long-term vitreous replacement. However, silicone oils have several disadvantages when they are employed as a VBS. These include the risk of secondary glaucoma, cataract formation, and the need for additional surgery in order to remove the silicone oil.

The human vitreous body is a viscoelastic gel with a viscosity of 4.2 g cm^3^/g and consists up to 98% of water and a matrix of hyaluronic acid (HA). Therefore, it seemed natural to develop VBS based on HA. Earlier studies using native hyaluronate (HA) in patients with retinal detachment (Pruett *et al*.*)* could only achieve a reattachment rate of about 18%, compared to 70–90% treated with silicone oil or gas tamponades [1, 8].The very short half-life of less than 14 days[9] of the non-crosslinked HA in the vitreous cavity was accounted for the unsatisfying results. An increase in intraocular pressure due to intensive swelling of the hydrogel was the most common side effect[8].

To prevent too rapid biodegradation, Spitzer et *al.[10]* and Schramm *et al*. applied chemically modified and UV cross-linked HA and demonstrated a good biocompatibility and a residence time of the hydrogel in the vitreous cavity of at least six weeks[11]. Moreover, the cross-linked hyaluronate hydrogels (CHA) remained in the vitreous cavity for several months. The complete biodegradation of the hydrogels occurred after six to nine months. No inflammatory or toxic reaction and no lens opacification were observed. The vitreous substitute had a refractive index of 1.338, which is very similar to that of the human vitreous and a clear advantage over silicone oils and gases. Similar results were observed by other authors using comparable CHA formulations[12]. However, all of the above-mentioned protocols used chemically cross-linked HA hydrogels. In contrast, the thiol-modified polymer of tVBS is able to build stable hydrogels by natural formation of disulfide bridges and thus does not require addition of chemical cross-linkers or other manipulation.

Apart from transparency, stability, sufficient viscosity, and biocompatibility, another practical advantage of the tVBS hydrogels are their hydrophilic properties in contrast to the hydrophobic silicone oils. The filling with both VBS is possible by simply injecting the hydrogel manually as well as by using a pump. These properties facilitate a complete fill of the vitreous cavity and a good tamponading effect.

The tVBS three-dimensional hydrogels combine many of the vitreous properties such as optical transparency, high viscosity, buoyancy and long-term stability as well as a good biocompatibility. However, the viscosity of our tVBS hydrogels was much higher that than of human vitreous. Lee et al. found the viscosity of healthy human vitreous to be 300–2000 cP (0.300 to 2 Pa·s).[13, 14] Thus, the e high viscosity of our tVBS hydrogels may be a suitable vitreous replacement in situations where a strong tamponading effect is desired.

The refractive index of tVBS hydrogels is similar to human vitreous or water.[15] This seems to be a common trait for hydrophilic hydrogels. This characteristic might be helpful in visual rehabilitation. In contrast, silicone oils have a refractive index of about 1.4 and induce a hyperopic shift of about 4–6 dioptres after surgery.[16]

For application during vitreoretinal surgery the crosslinked hydrogel must be injectable through a needle. The injection force required for instillation was comparable to standard ophthalmic viscosurgical devices (OVDs). Although injection of the hydrogels through a cannula reduces the viscosity of the tVBS, the rheological experiments showed that after an injection through a 20G or 23G needle, the viscosity at low shear rate remains high compared with silicone oil. The high viscosity of the crosslinked hydrogel may be important for the tamponading effect. Furthermore, a high viscosity may avoid turbulent flow in the area around a retinal hole and prevent re-detachment of the retina as well as secondary haemorrhage into the vitreous cavity.

Stability and persistence of a vitreous substitute within the vitreous cavity over a longer time period is of great importance, especially in the treatment of retinal detachments with a high risk for proliferative vitreoretinopathy (PVR). Further studies should investigate the in vivo degradation of the tVBS hydrogels, specifying their physical attributes at specific time points after instillation. The ERG in the VBS eyes both strong and soft formulation were slightly reduced despite of attached retinas. We believe that the reduction of the ERG that we saw in some animals after the operation is mainly due to vitrectomy. Consequently, we started long-term biocompatibility studies. The preliminary impression that we got so far is that the ERGs in animals that are filled with VBS after vitrectomy without inducing a retinal detachment are returning to baseline after about 4 weeks (data not shown).

We were surprised about the unexpectedly high rate of re-detachment in the silicone oil group. In humans, the rate usually is far lower.[17, 18] However, young rabbits have a strong predisposition for developing proliferative vitreoretinopathy and the success rate of silicone oil surgery for retinal detachments to our knowledge has not been evaluated to date. Moreover, we assume that the difference may be also inherent to the chosen rabbit model in which complete removal of the vitreous is much more difficult than in the adult human eye. The hydrophilic VBS may be more likely to fill the gaps of potential vitreous remnants than the hydrophobic silicone oil.

Vitrectomy and vitreous substitute especially when silicone is used often induce cataracts. Patients after vitrectomy often require early cataract surgery, which puts them at an additional surgical risk. Moreover, cataract surgery after vitrectomy is more prone to complication than cataract surgery in non-vitrectomized eyes. It was most remarkable that only a minority of eyes that received VBS soft (2 out of 7) or VBS strong (1 out of 8) developed cataracts.

## Conclusions

The results show the potential of two different tVBS formulations (VBS soft and VBS strong) for retinal detachment repair. In the presented study, the hydrogels were superior to silicone oil. Moreover, the hydrogels likely have additional advantages compared to the currently employed silicone oils, which require repeat surgery for oil removal. Such secondary procedures (with its inherent surgical risks of re-detachment, endophthalmitis, cataract development, hypotony, haemorrhage etc.) could be avoided when the self-degradable tVBS, strong or soft, would be used instead. The hydrogels were less frequently associated with cataract formation–a complication that is commonly observed for silicone oils and gases, which are the currently used vitreous substitutes.
